# The coming of age for branched-chain amino acids

**DOI:** 10.20517/jca.2021.02

**Published:** 2021-05-14

**Authors:** Chen Gao, Nancy Cao, Yibin Wang

**Affiliations:** Molecular Biology Institute, Department of Anesthesiology and Peri-operative Medicine, David Geffen School of Medicine, University of California, Los Angeles, CA 90095, USA.

In our ongoing quest to unlock the secrets for longevity and healthy aging, one of the most conserved and reproducible interventions proven to extend life-span across the evolutionary tree is dietary restriction^[1,2]^. Ample evidence has indicated that reduced food-intake significantly delays the onset of age dependent deterioration in neural, metabolic and cardiovascular systems and extend life-span from yeast, fly, rodents to non-human primates. Indeed, highly conserved nutrient sensing molecules and key metabolic regulators, such as mTOR, IGF1 and AMPK, have been implicated as the key players in the interaction between diet and health span^[3]^. An exciting progress in the field of aging studies is the emerging recognition that dietary restriction mediated benefits may be not due to the reduction of total calorie intake but rather resulted from restricted intake of specific macronutrient components. More specifically, protein restriction is shown to be responsible for the life-span extension effect of food/caloric restriction^[4]^. However, the precise components in dietary protein responsible for the life-span modulatory effect remain to be clearly defined. Recently, two studies from Lamming’s laboratory offered further evidence that branched-chain amino acids (BCAA consisting of valine, leucine and isoleucine) may hold the key to protein restriction mediated health benefits during aging^[5,6]^.

In the study by Richardson *et al.*^[6]^, two models of mutant mice with accelerated aging phenotype were first utilized. One is the *Lmna*^*G609G/G609G*^ mouse which carries the same disease-causing mutation in the *Lmna* gene for Hutchinson-Gilford Progeria Syndrome. Another is *Lmna*^*−/−*^ complete knockout mouse with complete deletion of the *Lmna* gene. Both mouse models developed progressive cardiomyopathy associated with accelerated aging. Authors treated these mice from weaning with either a low BCAA or a low total amino acids diet. The low BCAA diet treatment showed a significant extension in life span similar to the effect of low amino acid diet observed in female *Lmna*^*−/−*^ mice. A significant extension in life span was also made in both male and female *Lmna*^*G609G/G609G*^ mice. When implemented in wildtype mice, life-time feeding of a low BCAA diet extended healthy span (as measured by age-dependent changes in obesity and frailty index) in both male and female mice, but significantly extended life span only in male mice. In contrast, when low BCAA diet was initiated in mid-aged mice (16 months of age), aging related frailty was improved but no significant extension of life span was observed. These findings offered first *in vivo* evidence that a BCAA restricted diet might be sufficient to confer beneficial effect on healthy aging in mammals. Considering the known complications associated with low-protein diet in causing frailty and sarcopenia, results from Richardson *et al.*^[6]^ suggests that low-BCAA diet is able to prolong life-span while improving frailty and metabolic health in aged mice. This may have significant implications in future clinical translation. However, the study is still preliminary, and the benefits of low-BCAA diet are not yet fully characterized at organ physiology levels, including cardiovascular, respiratory, neural and metabolic systems. More importantly, the study also reveals the potential complications and limitations of BCAA targeted dietary manipulation. The low-BCAA diet mediated modulation on life-span appears to be influenced by sex but the mechanism is unknown^[6]^. In addition, low-BCAA diet failed to improve cardiac deterioration in the *Lmna*^−/−^ mice despite of a positive effect on life-span, suggesting that low-BCAA diet may affect other organs vital to longevity. Finally, none of the dietary manipulation was performed in mice with pre-existing conditions, such as cardiometabolic disorders. Therefore, low-BCAA mediated anti-aging effect is only demonstrated in a prophylactic manner and its application as a therapeutic strategy to halt aging process, particularly among vulnerable populations with pre-existing mortality risks remain to be demonstrated.

At mechanistic level, the anti-aging effect of low-BCAA diet highlights the importance of BCAA as critical nutrients as well as potent nutrient signal molecules for organ function and homeostasis. Indeed, BCAA have unique contribution to nutrient signaling relative to all other essential amino acids^[7]^. BCAA is known to link nutrient environment with cellular growth and metabolism through well established mTOR and other signaling pathways. Therefore, it is actually quite assuring that through unbiased transcriptome profiling in skeletal muscle tissue, authors indeed found that the molecular signatures associated with different BCAA treatment groups are highly enriched with genes involved in mTOR mediated downstream signaling pathways, such as phagosome function (for autophagy) and PI3K-AKT pathway^[6]^.

Giving the potential impact of BCAA restricted diet in aging modulation, and next obvious question is which of the three BCAAs are responsible for the observed effect. BCAA are essential amino acid with three members, valine, leucine and isoleucine. Because they share the same rate-limiting enzymes as part of their individual catabolic pathways, the homeostatic regulation of the three BCAA is often intricately correlated. However, extensive evidence suggests each of the BCAA may have unique and distinct function in metabolic regulation and nutrient signaling. In a subsequent study by the same group, Yu *et al.*^[5]^ further delineated the specific role of each individual BCAA in dietary impact on metabolic health.

Using a series of amino acid defined diets, Yu *et al.*^[5]^ systematically measured the impact of lowering individual BCAA intake on the metabolic health in mice. While lowering BCAA as a group showed potent and unique effect to improve metabolic status comparing to other essential non-BCAA amino acids, lowering individual BCAA in food intake revealed a strong effect of valine and isoleucine on glucose control, insulin resistance and hepatic gluconeogenesis. In contrast, lowering leucine showed no effect on the same metabolic indexes. Conversely, when individual BCAA was added back to the low-amino acid diet, isoleucine and valine were able to at least in part abolish the beneficial effects conferred by low-amino acid diet. Again, adding back leucine did not have such effect. These results suggest that isoleucine and valine are potentially responsible for the metabolic outcome from BCAA manipulation, instead of leucine. Such a revelation is somewhat unexpected, but consistent with the observation made in liver-specific *Tsc1* knockout and *Gcn2* knockout mice by the authors. Yu *et al.*^[5]^ demonstrated that isoleucine restriction mediated beneficial effects were not affected in these mice, implicating an mTOR and Gcn2 independent mechanism, both pathways are known mediators of leucine induced signaling in cells. Instead, from transcriptome analysis in liver and adipose tissues, authors identified a significant perturbation in *FGF21* expression and energy expenditure following isoleucine restriction, which was necessary for low-isoleucine diet mediated metabolic protection in mice. Therefore, as nutrient signaling molecules, individual BCAA exerts distinct downstream activity in metabolically critical tissues, such as liver, muscle and fat.

Although many questions still remain to be answered for the physiological importance and molecular mechanisms in BCAA mediated modulation in aging, these recent findings continue to foster the increasing recognition that BCAA homeostasis and regulation are central to health span and longevity [Figure 1]. BCAA is still widely promoted as a healthy and beneficial dietary supplement to help sarcopenia and metabolic disorder, particularly in aged population. Yet, scientific evidence supporting such benefits remains controversial. Our past view on BCAA as a single group of nutrient source should also be reexamined in light of these findings on the differential impact of individual BCAAs. More studies, like these recent reports from Lamming’s laboratory, are critically needed to understand the fundamental mechanisms involved in BCAA mediated cellular signaling in specific tissues in order to decipher the molecular players involved in the beneficial *vs*. deleterious effects of BCAA in aging process. These insights will not only provide vital information for future development of anti-aging mimics to combat age related diseases, but may also raise necessary awareness to the dietary intake of BCAA from our daily meals.

## Figures and Tables

**Figure 1.**
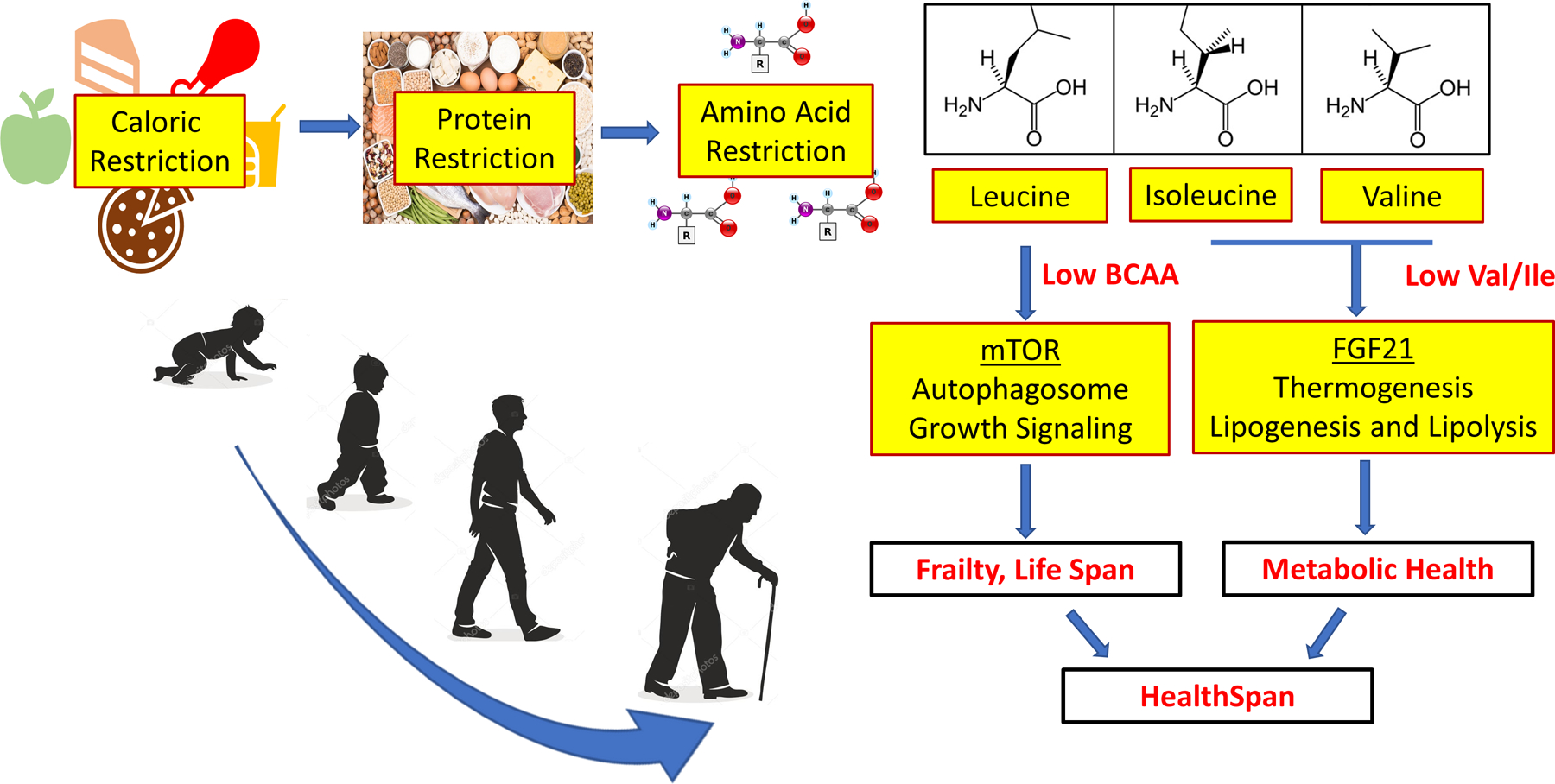
BCAA restricted diet in healthspan modulation.
